# Why do attention‐deficit/hyperactive disorder and/or autism traits place adolescents at risk for depression? Protocol for a longitudinal comparison of the mediating role of emotion regulation deficits versus emotional burden

**DOI:** 10.1002/jcv2.70052

**Published:** 2025-10-28

**Authors:** Edmund J. S. Sonuga‐Barke, Melanie Palmer, Kirsty Griffiths, Anna Wyatt, Andrea Danese, Susie Chandler, Daniel Stahl, Steve Lukito, Georgia Pavlopoulou, Emily Simonoff, the RE‐STAR team

**Affiliations:** ^1^ Department of Child & Adolescent Psychiatry Institute of Psychiatry, Psychology & Neuroscience King's College London London UK; ^2^ Department of Child and Adolescent Psychiatry Aarhus University Aarhus Denmark; ^3^ Department of Psychology Hong Kong University Hong Kong China; ^4^ University College London London UK; ^5^ Department of Biostatistics and Health Informatics Institute of Psychiatry Psychology & Neuroscience King's College London London UK; ^6^ Anna Freud Centre London UK; ^7^ South London and Maudsley NHS Trust London UK

**Keywords:** ADHD, adolescence, autism, longitudinal, mental health, schools

## Abstract

**Background:**

Depression levels increase dramatically during adolescence in the general population. This effect is exacerbated in adolescents with a diagnosis of attention‐deficit/hyperactivity disorder (ADHD), autism, or both. Here we detail the protocol for the *My Emotions and Me Over Time* (MEMO) study, a 12‐month longitudinal study with the primary aim to compare two competing hypotheses for why this is the case. The first, established hypothesis is that depression risk associated with ADHD and/or autism is mediated by emotion regulation deficits (ERD). The second and new perspective is that it is mediated by the experience of elevated *emotional burden* (EB) created by (i) greater exposure to upsetting events and encounters, which are (ii) experienced more intensely. Cross‐lagged path models will test the relative importance of the ERD and EB pathways to the relationship between autism and ADHD traits and depression symptoms. Exploratory analyses examining secondary mediators (i.e., self‐esteem) and moderators (rumination, resilience and alexithymia) will also be conducted.

**Methods:**

A sample of 600 adolescents aged 11–16 years, enriched for the presence of autism and ADHD diagnosed cases, and their parent/guardian will be recruited via schools, local NHS (South London and Maudsley) and ADHD and autism charities. Measures of ADHD, autism and depression, ERD, EB, self‐esteem, rumination, resilience and alexithymia will be completed at baseline, 6 months and/or 12 months by parents and/or participants. Background factors such as age, sex, cognitive abilities and socioeconomic status as well as service use and medication status will also be collected.

**Results:**

The pathways between ADHD/autism and depression, along with their mediators and moderators, will be analysed using structural equation modelling.

**Conclusion:**

The findings from MEMO will feed into the other studies within the RE‐STAR programme to support the development of an intervention to reduce ADHD and/or autism‐related depression risk in adolescence.

## Key Points

**What's known:**

- The pathway between neurodivergence and depression has been shown to be mediated by emotion dysregulation (ED).
- Currently interpreting this evidence to illuminate implication for clinical translation is hampered by ambiguities and vagueness in the definition of ED.

**What's new:**

- The *My Emotions and Me Over Time* (MEMO) study is designed to test the mediating role of two distinct ED pathways to depression in neurodivergent people: (i) Emotion Regulation Deficits (ERD) and (ii) Emotional Burden (EB).
- The latter bringing a novel perspective focusing on the frequency and intensity of upsetting experiences in driving depression risk.

**Clinical implications:**

- By parsing different ED constructs, MEMO may help identify new intervention targets.

## Graphical abstract

This paper details the protocol for the My Emotions and Me Over Time (MEMO) study, a longitudinal study exploring why ADHD and/or autism traits place adolescents at greater risk for depression. This study will explore two potential mediating factors in pathways to depression: emotional regulation deficits; and emotional burden.

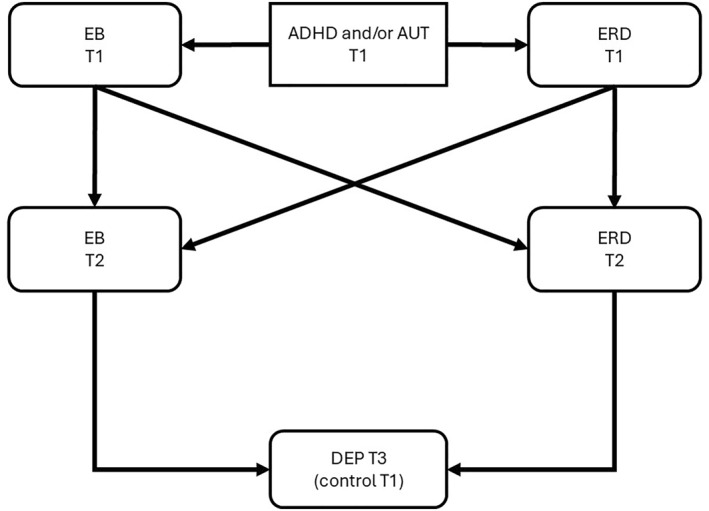

## INTRODUCTION

Rates of depression increase dramatically across the adolescent period in the general population (Shore et al., 2018). This creates substantial distress and impairment that can persist life‐long (Balázs et al., 2013). Attention‐deficit/hyperactivity disorder (ADHD) and/or autism both exacerbate this risk substantially. By adulthood around 50% of people with autism (Dow et al., 2021; Hollocks et al., 2019), and 30% of those with ADHD, will have a clinical depression diagnosis (Wilens et al., 2009). This risk is also present, though to a lesser degree, in those with elevated sub‐clinical symptoms. It is greater where ADHD and autism co‐occur (Factor et al., 2017), which they commonly do (Kushki et al., 2019). Preventative interventions focusing on reducing depression risk, adapted to the needs of young people with ADHD and autism, are urgently required. To build such interventions we need to first understand why these neurodivergent young people develop depression (Keck et al., 2018). In this protocol we describe a longitudinal study, *My Emotions and Me Over Time* (MEMO), a part of the *Regulating Emotions—Strengthening Adolescent Resilience* (RE‐STAR) programme (https://www.kcl.ac.uk/research/re‐star), designed to compare two different hypotheses about emotion‐related mediators of the path between neurodivergence and adolescent depression.

The emotion regulation deficit (ERD) hypothesis builds on the influential view that depression emerges during adolescence in individuals with ADHD and autism, in part, because of an impaired ability to regulate their negative emotions (e.g., anger, frustration, sadness, etc.), caused by cognitive dysfunction *within* the individual. That emotion regulation deficits of this sort increases the risk of the emergence of depression in adolescence, in general, is well established (Durbin & Shafir, 2008). That young people with ADHD (P. Shaw et al., 2014) and/or autism (McDonald et al., 2024) display elevated emotional lability, irritability and reactivity understood as signal markers of such deficits, is supported by findings from countless studies (e.g., Bunford et al., 2015, 2018; Eyre et al., 2019; Sáez‐Suanes & Álvarez‐Couto, 2022; Seymour et al., 2012, 2014). Furthermore, these expressions of impaired emotion regulation in autism and/or ADHD have been linked to alterations at multiple cognitive levels, including emotion perception (Krause et al., 2021; Kret & Ploeger, 2015; Oakley et al., 2022) and awareness (Bunford et al., 2015; Conner et al., 2019; Huggins et al., 2021; Roberts‐Collins et al., 2018); executive control of emotional impulses (Biederman et al., 2012; Seymour et al., 2014); and meta‐cognitive processes (Muniandy et al., 2023; Pouw et al., 2013). More direct evidence for the mediating role of emotion regulation deficits in driving depression risk in ADHD and autism, more specifically comes from multiple longitudinal studies. Seymour et al. (2012) demonstrated that difficulties in emotion regulation fully mediated the longitudinal relationship between ADHD and depression. Similar results have been seen for autism, for instance, Barnes et al. (2025) showing how emotion regulation deficits mediate the pathway from elements of autism such as intolerance of uncertainty and depression. In MEMO, ERD will be measured using the *Difficulties in Emotion Regulation Scale* (Victor & Klonsky, 2016). We chose this as our core ERD measure for a number of reasons. First, it focuses on the regulation of negative emotion which aligns with MEMO's goals. Second, it offers a broad coverage of the ERD concept, covering six domains with separate subscales: lack of emotional awareness; lack of emotional clarity; nonacceptance of emotional responses; difficulty engaging in goal‐directed behaviour; impulse control difficulties; limited access to emotion regulation strategies. Third, because it has been employed as a measure of ERD in studies of autism and ADHD and has shown strong psychometric properties when used in samples of young people with ADHD (Bunford et al., 2020) and autism (McVey et al., 2022). It is important to note, however, that the DERS represents one of several conceptualisations of ERD and measures specific facets of the broader construct. Our findings will therefore be most directly relevant to ERD as operationalised within the DERS framework and should not be assumed to generalise to all manifestations of emotion regulation difficulties.

In contrast, the emotional burden (EB) hypothesis provides an explanation of depression risk in young people with autism and ADHD, not in terms of regulatory deficits within the individual but rather to their experiences of *extrinsic adverse events* and encounters in their everyday lives. In this way it is more aligned to a neurodiversity than a disorder paradigm (Sonuga‐Barke, 2023). The EB formulation has emerged from within RE‐STAR. First, qualitative analysis of neurodivergent young people's accounts obtained during interviews have highlighted how powerfully negative emotions induced by common upsetting events (CUEs), are experienced by young people with ADHD and autism, and how much these impacted their lives (Pavlopoulou et al., in press). Second, *the quantitative observation*, *based on data from a new questionnaire*, *the My Emotions in School Inventory* (Lukito et al., in press) designed specifically to explore this observation further, in that ADHD and autism both dramatically increase the *frequency* with which individuals are exposed to CUEs in school, identified in the interviews as a particularly significant source of EB, and the *intensity* with which they are experienced (Eccleston et al., 2019; Mansfield & Soni, 2024). These observations align to prior research showing that individuals with ADHD and autism are exposed to elevated levels of daily hassles, stressors and negative life events, compared to neurotypical individuals (Eccleston et al., 2019; Taylor & Gotham, 2016), and that certain exposures have heightened salience for them (Beck et al., 2024; Rumball et al., 2020, 2021). Based on these two elements, we have operationalised EB as a product of the frequency × intensity with which CUEs are experienced (Lukito et al., in press).

The MESI was co‐developed with young people with ADHD and/or autism based following extensive interviews (Pavlopoulou et al., 2025). It asks about the experience of 25 school‐based CUEs. Initial findings support the psychometric strength of the instrument and its power to differentiate neurodivergent from neurotypical adolescents. And predict depression over and above, its association with ERDs as measured by the DERS (Lukito et al., in press).

MEMO will test whether autism and/or ADHD are associated with increases in depression symptoms during adolescence, and if so whether the changes are mediated by ERD and/or EB. This protocol describes short‐term changes in depression symptom trajectories over 12‐month periods in a sample of adolescents between the ages of 11 and 16, stratified by age, enriched for the presence of ADHD and autism diagnoses. Measures of ADHD, autism and depression, ERD, EB, self‐esteem, rumination, resilience and alexithymia will be completed at baseline (T1), 6 months (T2) and/or 12 months (T3) by parents and/or young people. Prior studies have demonstrated that in high‐risk groups, increases in depression can be identified, and their predictors assessed over 12 months (e.g., Rice et al., 2019). This is consistent with the view that adolescences is a period of developmental flux where potentially vulnerable individuals (e.g., those with ADHD and autism) might be especially likely to show changes in symptomatology. The expectation is that MEMO will be extended to cover medium and long‐term trajectories in future protocols.

### Research questions

For all questions ADHD, autism and depression, as well as other mediators, moderators and other outcomes will first be modelled as continuous variables in all models. Non‐linear effects will then be explored using ADHD and autism diagnostic status as binary predictor variables.

**FIGURE 1:**
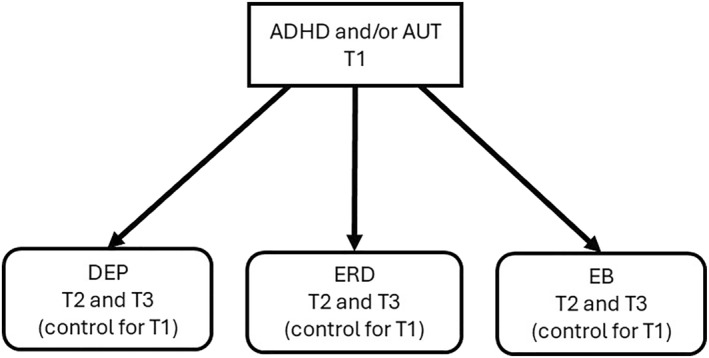
Schematic of the first hypothesis to be tested in MEMO. All figures simplified to illustrate hypothesis testing. ADHD, attention‐deficit/hyperactivity disorder symptoms; AUT, autism symptoms; EB, emotional burden; ERD, emotion regulation deficits; DEP, depression.

**FIGURE 2:**
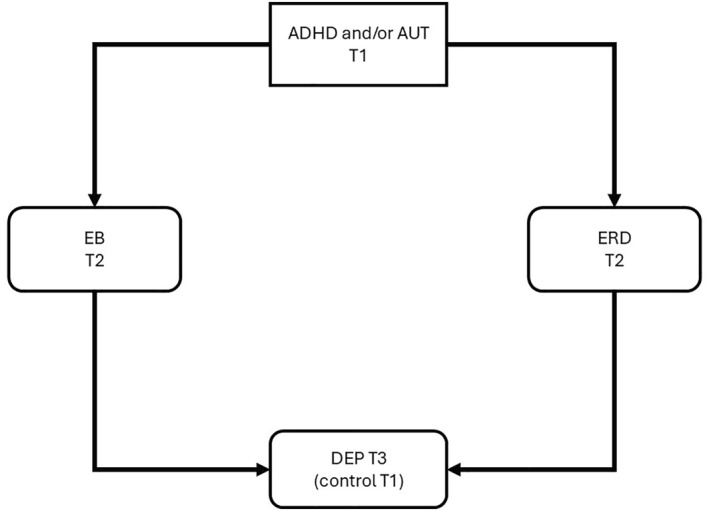
Schematic of the core mediational model to be tested in MEMO. All figures simplified to illustrate hypothesis testing. ADHD, attention‐deficit/hyperactivity disorder symptoms; AUT, autism symptoms; EB, emotional burden; ERD, emotion regulation deficits; DEP, depression.

**FIGURE 3:**
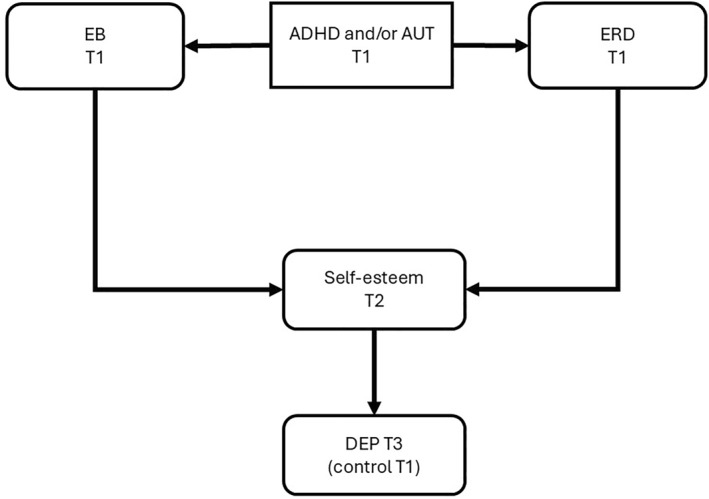
Schematic of the model with a secondary mediator (self‐esteem) to be tested in MEMO. All figures simplified to illustrate hypothesis testing. ADHD, attention‐deficit/hyperactivity disorder symptoms; AUT, autism symptoms; EB, emotional burden; ERD, emotion regulation deficits; DEP, depression.

**FIGURE 4:**
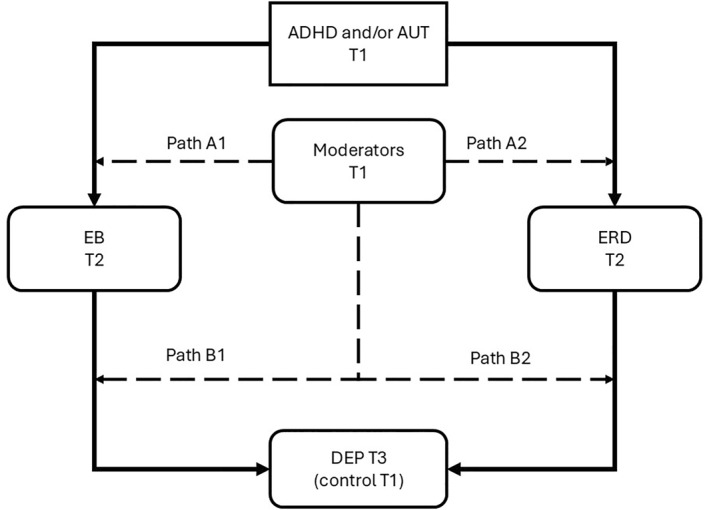
Schematic of the model with moderators that will be tested in MEMO. All figures simplified to highlight hypothesis testing. Path A, the moderator analysis testing the pathway between ADHD and AUT to ERD and/or EB. Path B, the moderator analysis testing the pathway between ERD and/or EB to depression symptoms. ADHD, attention‐deficit/hyperactivity disorder symptoms; AUT, autism symptoms; EB, emotional burden; ERD, emotion regulation deficits; DEP, depression.

**FIGURE 5:**
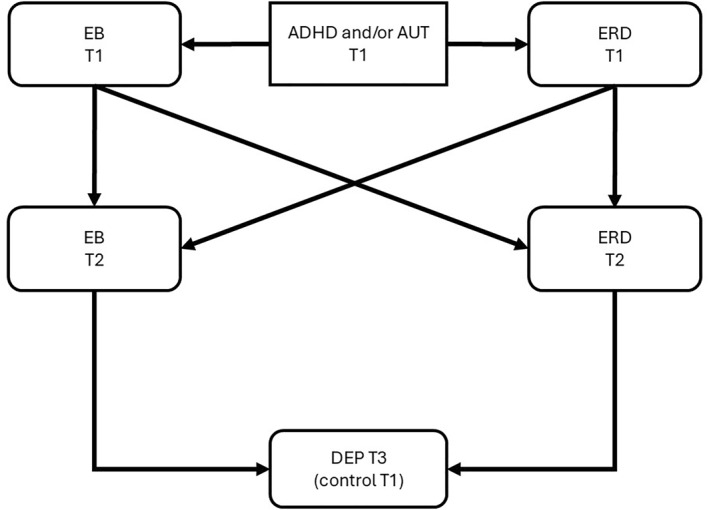
Schematic of the model of the temporal relationship between EB, ERD and depression that will be tested in MEMO. All figures simplified to illustrate hypothesis testing. ADHD, attention‐deficit/hyperactivity disorder symptoms; AUT, autism symptoms; EB, emotional burden; ERD, emotion regulation deficits; DEP, depression.

The hypothesised paths are illustrated in Figures 1, 2, 3, 4, 5. Please note the figures are intended to display only the hypothesised paths relevant to each research question. For clarity, we omit specific reference to time points and the autoregressive effects and other paths, including confounders, which are included in the statistical models but not shown visually.

#### Primary research questions (1–2)

Q 1 Are ADHD and/or autism traits (T1) associated with later depression symptoms and ERD and/or EB at T2 and T3, controlling for baseline outcome levels?

The model to be tested is illustrated in Figure 1.

Q 2 Do ERD and/or EB (T2) mediate the developmental association between ADHD and/or autism (T1) and depression symptoms (T3)?

Figure 2 presents the mediational model that will be tested. Covariates will include a range of background and child‐related factors.

#### Exploratory research questions (3–6)

Q 3 Are depression risk pathways from ERD and/or EB associated with ADHD/autism secondarily mediated by self‐esteem?

Self‐esteem has been selected as the secondary mediator because low self‐esteem is associated with ADHD and autism traits (Bettancourt et al., 2024; McCauley et al., 2019) and is also an established risk marker for emergent depression (Cong et al., 2021; Gittins & Hunt, 2020; van der Cruijsen & Boyer, 2021) potentially representing a precursor of depression that could help target prevention efforts (Mann et al., 2004). Figure 3 illustrates the model that will be tested. Assuming that ADHD and autism are stable traits, an assumption that will be tested in the study by assessing auto‐regression from T1 to T3, and with only three assessment points, for this analysis we will include ADHD/autism and ERD and EB measures collected at T1. The secondary mediator self‐esteem will be measured at T2, and the outcome, depression, will be measured at T3.

Q 4 Are pathways from ERD and/or EB associated with ADHD/autism to adolescent depression risk moderated by key psychological traits?

We will test the moderating effects of three psychological traits, previously shown to be associated with emotion regulation and/or depression risk in neurodivergent individuals, but also known to *vary between* neurodivergent young people: (i) personal and interpersonal resilience levels (Black et al., 2024; Kuenzel & Duerden, 2024); (ii) the tendency to ruminate (Harmon et al., 2020) and (iii) alexithymia (difficulty experiencing, identifying, and expressing emotions) (Edel et al., 2010; Oakley et al., 2022). These three moderators were selected because of their theoretical relevance to emotional regulation processes. Rumination, a repetitive focus on distress, is linked to sustained negative affect and depression onset (Nolen‐Hoeksema et al., 2008) and may exacerbate ERD impacts in ADHD/autism. Resilience reflects adaptive capacity in the face of adversity and may buffer the effects of ERD and EB on depression risk (Fergus & Zimmerman, 2005). Alexithymia, characterised by difficulties identifying and describing emotions, is highly prevalent in autism and ADHD (Kinnaird et al., 2019) and associated with poor emotion regulation and heightened depression risk (Preece et al., 2022). Figure 4 illustrates the moderator analyses.

The extent to which moderation will occur between ADHD traits, autism, and EB/ERD and/or after them will be tested. In a similar test to our primary research questions, we will assess ADHD and/or autism at T1, EB and ERD as mediators at T2, and depression at T3. The moderators will be measured at T1 as they have been shown to be stable over time (Blanke et al., 2023; Hankin et al., 2015; Z. Shaw et al., 2019). Effects of the moderators will be assessed on pathways leading to and from the mediators.

Q 5 Do EB and ERD influence each other reciprocally over time?

While ERD is typically considered as an early emerging intrinsic characteristic of the individual (Borelli et al., 2017; Castellini et al., 2023; Hawn et al., 2015; Nigg et al., 2020), it may also emerge later in development following exposure to adverse social experiences (e.g., CUEs) (Gruhn & Compas, 2020; Miu et al., 2022). In this study, we will explore this by testing the possibility that EB (linked to CUE exposure) will drive ERD over time. The reciprocal pathway from ERD‐to‐EB will also be explored. To investigate this, we will introduce additional pathways: from EB at T1 to ERD at T2 to T3 depression, and from ERD at T1 to EB at T2 to T3 depression. Figure 5 illustrates the structure of such an analysis.

Q 6 Are effects specific to depression, or do they extend to anxiety and oppositionality/defiance as an outcome?

To assess whether the findings will be specific to self‐reported depression symptoms, the same analyses will be conducted with parent‐rated depression and anxiety as the T3 outcome as part of the exploratory analysis. While depression and anxiety are distinct in their clinical presentations—depression often marked by low mood, and anxiety by excessive worry—these conditions share common underlying mechanisms (Eysenck & Fajkowska, 2018; Kaiser et al., 2021). Likewise, ADHD and oppositionality/defiance are highly comorbid and may share aetiological elements in common (Azeredo et al., 2018). By including anxiety and oppositionality/defiance as secondary outcomes, the analysis will acknowledge these overlapping processes while allowing for separate assessment of each condition's unique contributions.

## MATERIALS AND METHODS

### Design

The initial phase of MEMO covered in this protocol will be a 12‐month longitudinal study, with data to be collected at three timepoints: 0 months (T1), 6‐month (T2), and 12‐month (T3). Parents/guardian and young people will complete questionnaires using an online platform called Qualtrics^XM^ (Provo, UT). A Design Summary table is included in the Supporting Information S1, mapping the primary research questions to their hypotheses, sampling plan and analyses, potential outcomes and interpretations.

### Participants

The sample will consist of 600 adolescents (aged 11 and 16 years) and their parents/guardians. This includes 160 individuals with ADHD and/or autism diagnosis and 440 from mainstream secondary schools, balanced, as far as possible by year group (Year 7–11) and sex. All participants must attend mainstream schools; those in Special Educational Needs (SEN) schools or in a Pupil Referral Unit for more than one academic term will not be invited to participate.

Recruitment will occur through mainstream secondary schools (i.e., school sample) and local NHS and national autism and ADHD charities (i.e., clinical sample). Diagnostic records will be accessed through NHS Trusts. Individuals with intellectual disabilities (ICD‐10, F70‐F74) will not be invited to participate. Participants recruited from charities will provide documentary proof of diagnosis. All participants will be English‐proficient to complete the online questionnaires. See Table 1 for the recruitment numbers for school and clinical samples.

**TABLE 1:** Proposed sample size for MEMO study, split by year group (Year 7–11) and by recruitment pathway (clinical or school sample).

	Sample		Total
	Clinical	School	
Year 7 (ages 11/12)	40	110	150
Year 8 (ages 12/13)	40	110	150
Year 9 (ages 13/14)	40	110	150
Years 10 and 11 (ages 14/15/16)	40	110	150
Total	160	440	600

### Measures

The measures described below will be used to answer the research questions set out above. Additional measures collected in MEMO, for additional analyses not related to the above core research questions, are described in the Supporting Information S1: Supporting Information I.

### Demographics

Demographic information will be collected from parents/guardians (child's age, sex, ethnicity, diagnosis, family history of neurodevelopmental/psychiatric conditions, service use, whether or not medication has been prescribed for mental health and neurodevelopmental conditions (and what type), parents/guardian's education and employment status). Postcode will be converted into a household deprivation index, serving as our measure of SES. Schools will provide information on the child's free school meal eligibility and current SEN support.

### School characteristics

The Department of Education will provide data on a school's Ofsted rating, percentage of pupils receiving free school meals, percentage receiving SEN support, percentage with an SEN Education, Health and Care Plan, and percentage whose first language is not English. Schools' absence record and attainment scores will also be collected.

### Predictors

#### ADHD symptoms

The Swanson, Nolan and Pelham Rating Scale Teacher and Parent‐rated questionnaires (SNAP‐IV) (Swanson, 1992) uses a 4‐point Likert scale, ranging from *Not at all* (1) to *Very much* (4). Scores will be calculated by totalling the scores for items in each subscale and dividing by the number of items, with higher scores indicating greater ADHD traits. The combined score of the Inattention and Hyperactivity/Impulsivity subscales will serve as the measure of ADHD traits.

#### Autism symptoms

The Social Communication Questionnaire (SCQ‐current; Rutter, 2003) is a 40‐item parent‐report measure of autism symptomatology suitable for both verbal and non‐verbal children. The items require a dichotomous Yes (1) or No (0) response about the child's capabilities over the past 3 months. Total scores range from 0 to 39, with higher scores indicating greater symptoms of autism (Berument et al., 1999). The SCQ has shown strong psychometric properties in a range of different populations (Chesnut et al., 2017) including male and female samples (e.g., Evans et al., 2019).

### Outcomes

#### Primary outcome

##### Child‐rated depression symptoms

The Mood and Feelings Questionnaire (MFQ; Angold et al., 1995) is a 13‐item measure for assessing depressive symptoms. It has a 3‐point scale from Not at all (0) to True (2). Total scores range from 0 to 26, with higher scores indicating a greater risk of depressive symptom.

#### Secondary outcomes

##### Parent‐rated depression symptoms

The parent‐rated MFQ version is a companion to the child version. The correlation between parent and child is *r* = 0.51–0.53 (Jarbin et al., 2020).

##### Anxiety symptoms

The Spence Children's Anxiety Screen (SCAS‐8; Reardon et al., 2018) is an eight‐item self‐report questionnaire to be completed by young people. The SCAS‐8 items will ask about a range of anxiety disorders, with items rated on a 4‐point scale ranging from *Never* (0) to *Always* (3). SCAS‐8 scores will range between 0 and 24, with higher scores indicating higher levels of anxiety symptoms.

### Mediators

#### Primary mediators

##### Emotional burden

The *My Emotions in School Index* (MESI; Lukito et al., in press) is a 25‐item self‐report questionnaire about a young person's CUEs in school (see Supporting Information S1: Supporting Information II). The MESI was developed with input from RE‐STAR's youth panel who have a diagnosis of ADHD and/or Autism (Pavlopoulou et al., 2025). The MESI will evaluate the emotional experiences of 25 common events in schools, asking young people to rate the frequency and intensity of CUEs. The frequency of a CUE will be measured on a 5‐point scale, ranging from *Never* (0) to *Frequently* (4). The emotional intensity of the CUE is measured on a 5‐point scale, ranging from *Not at all* (0) to *Extremely* (4). EB will be calculated by multiplying frequency and intensity scores, with higher scores indicating greater EB. The MESI has high internal and test‐re‐test reliability and is associated with anxiety and depression, independently of ERD (Lukito et al., in press).

##### Emotion regulation difficulties

The *Difficulties in Emotion Regulation Scale* (DERS‐18; Victor & Klonsky, 2016) is an 18‐item self‐report measure to be completed by the young person and is an abbreviated version of the original 36‐item DERS (Gratz & Roemer, 2004). The DERS‐18 is composed of the three highest‐loaded items in each of the six subscales from the original 36‐item DERS: lack of emotional awareness, lack of clarity, non‐acceptance of one's emotions, difficulty engaging in goal‐directed behaviour, impulse control difficulties and lack of access to effective strategies. The DERS‐18 will be measured on a 5‐point frequency scales, ranging from Almost Never (1) to Almost always (5). The total DERS‐18 score ranges between 18 and 80, with higher scores reflecting greater emotion regulation difficulties. While these domains provide a robust operationalisation of ERD for the purposes of MEMO, they reflect one specific conceptualisation of emotion regulation difficulties. As such, the study's findings are interpreted in the context of these domains rather than the full range of processes encompassed by the broader ERD construct.

#### Secondary mediator

##### Self‐esteem

The Rosenberg Self Esteem Scale (RSE; Rosenberg, 1979) is a 10‐item self‐report measure to be completed by young people. The RSE measures overall self‐worth and is rated on a 4‐point scale, ranging from *Strongly disagree* (0) to *Strongly agree* (3). Score range from 0 to 30, with higher scores indicating greater self‐worth and positive self‐image.

### Moderators

#### Tendency to ruminate

The short‐version Rumination Response Style (RRS; Treynor et al., 2003) is a 10‐item self‐report measure to be completed by young people. The RRS assesses rumination styles, such as brooding and reflective pondering, using a 4‐point scale ranging from *Almost Never* (1) to *Almost always* (4). Total RRS scores range from 10 to 40, with higher scores representing greater levels of rumination.

#### Resilience

The Child and Youth Resilience Measure (CYRM; Jefferies et al., 2019) is a 17‐item self‐report measure that will assess social‐ecological resilience across individual, relational, communal, and cultural factors in young people. Responses will be recorded on a 5‐point Likert scale, ranging from *Not at All* (1) to *A lot* (5). Total resilience scores will range from 17 to 85, with higher scores indicating better access to personal, relational, and community resources, reflecting greater overall mental resilience.

#### Alexithymia

The Toronto Alexithymia Scale (TAS‐20; Leising et al., 2009) is a 20‐item self‐report measure to be completed by young people that measure difficulties in identifying and describing emotions. TAS‐20 items will be rated on a 5‐point Likert scale, ranging from *Strongly Disagree* (1) to *Strongly Agree* (5). Total scores will range from 20 to 60, with higher scores indicating a greater likelihood of alexithymia.

### Covariates

#### Demographic information

Socioeconomic status (index of multiple deprivation converted from parent‐provided postcode), will be included as a covariate.

#### Cognitive abilities

The young person's Cognitive Abilities Test (CAT4) score provided from schools will be the measure of cognitive ability. CAT4 scores have been demonstrated to be highly correlated with standardised full‐scale IQ scores (*r* = 0.76) (Simonoff et al., 2006).

The timing of the collections for different measures (T1, T2, and T3) are summarised in Table 2.

**TABLE 2:** Schedule of assessments across the three MEMO timepoints: 0‐month (T1), 6 months (T2), and 12 months (T3).

	Concept	Reason for inclusion	Measure	Timepoint		
				0‐month	6‐months	12‐months
				(T1)	(T2)	(T3)
Parent rated	Family background	Sample description, co‐variates	Demographics	X		
	Young person medication status	Sample description, sensitivity analysis	Background questions	X		
	Young people service use	Sample description	Service use	X	X	X
	Young person ADHD traits	Sample description, predictor	SNAP‐IV	X		X
	Young person autism traits	Sample description, predictor	SCQ‐current	X		X
	Young person depression	Outcome, risk factor	MFQ	X	X	X
	Young person anxiety	Outcome, risk factor	SCAS‐8	X	X	X
	Young person oppositional defiance disorder symptoms	Sample description	SNAP‐IV	X		X
	Young person emotional regulation	Moderator	DERS‐18	X	X	X
	Parental view of engagement and confidence in school	Exploratory	WOSP	X		
Young person rated	Family background	Sample description, co‐variates	Demographics	X		
	About my school	Sample description	About school	X	X	X
	Depression	Outcome, risk factor	MFQ	X	X	X
	Depression	Outcome, risk factor	PHQ‐A	X		X
	Anxiety	Outcome, risk factor	SCAS‐8	X	X	X
	Anxiety	Outcome, risk factor	GAD‐7	X		X
	PTSD	Exploratory	CPSS‐SF	X	X	X
	Daily stressors and hassles	Exploratory	APES	X	X	X
	Digital stressors and hassles	Exploratory	DAFI section A	X	X	X
	Emotional burden	Mediator	MESI	X	X	X
	Emotional regulation deficits	Mediator	DERS‐18	X	X	X
	Self‐esteem	Mediator	RSE	X	X	X
	Positive wellbeing	Exploratory	SWEMWBS	X	X	X
	Loneliness	Exploratory	UCLA	X	X	X
	Resilience, support (peer and familial)	Moderator	CYRM	X		
	Rumination	Moderator	RRS‐10	X	X	X
	Alexithymia	Moderator	TAS	X		
School‐based measures	Cognitive ability	Sample description, co‐variate	CAT	X		
	School policies	Sample description	Ofsted reports and school policy documents	X		

Abbreviations: APES, Adolescent Perceived Events Scale; CAT, Cognitive Abilities Test; CPSS‐SR, Child PTSD Symptoms Scale self‐report; CYRM, Child and Youth Resilience Measure; DAFI, Digital Activity; Feelings and Impact; DERS, Difficulties in Emotion Regulation Scale; GAD‐7, Generalised Anxiety Disorder; MESI, My Emotions in School Index; MFQ, Mood and Feelings Questionnaire; PHQ‐A, Patient Health Questionnaire‐Adolescent; RQ, Research Question; RRS, Rumination Response Style; SCAS, Spence Children's Anxiety Scale; SCQ, Social Communication Questionnaire; RSE, Rosenberg Self Esteem Scale; SNAP‐IV, Swanson, Nolan and Pelham Teacher and Parent Rating Scale; SWEMWBS, Short Warwick Edinburgh Mental Wellbeing Scale; TAS, Toronto Alexithymia Scale; WOSP, Wider Outcomes Survey for Parents.

### Procedure

The study has been approved by the Health and Social Care Research Ethics Committee of King's College London (IRAS number 327207).

### School recruitment

Mainstream secondary schools will be engaged through King's Widening Participation team and RE‐STAR's partner, Place2Be. Once a school has expressed an interest, the research team meet the school and present an overview of MEMO plus the benefits of partnering (e.g., free workshops and assembly presentations). After the headteacher consents a recruitment support pack will be sent, including an electronic flyer for school newsletters. The research team may also distribute physical flyers to schools. Recruitment rates will be monitored, and weekly summarises sent to the school's key contact. Parent/guardians will provide consent via the survey link on the flyer, and the child's assent will be required before receiving the T1 survey.

### Clinical recruitment

The clinical sample will be recruited through *South London and Maudsley NHS Foundation Trust* (SLaM) using the “Consent for Contact” mechanism linked to the Clinical Record Interactive Search. This process will mirror RE‐STAR's Work Package 1 (IRAS number 307053 and 319593). Young people with a clinical diagnosis of ADHD and/or autism will be recruited through charities (e.g., ADHD Foundation and Autistica), with proof of diagnosis required from the parent/guardian in the form of an original version of a letter from a clinician confirming the relevant diagnosis. Participants from previous studies who consented to future contact will also be approached. A telephone screening will assess eligibility, and eligible participants will receive the survey link via email.

### Online data collection

Data will be collected using Qualtrics (https://www.qualtrics.com), an online survey platform, which participants will be able to access via computer, laptop or smartphone. Surveys will be administered at three timepoints: baseline (T1), 6 months (T2), and 12 months (T3) from the date of consent. At each timepoint, participants will receive detailed instructions on how to complete the surveys.

Parents/guardians will be directed to an online information sheet and consent form upon accessing the invitation link (shared in the school's weekly e‐newsletters). Consent will be provided by selecting checkboxes and submitting an e‐signature. Parents/guardians can download these documents or request copies from the research team. After consenting and providing the young person's contact information, Qualtrics will send an invitation link to the young person, along with an adapted information sheet. The young person will provide assent by completing an online assent form with an e‐signature. Once both parental consent and young person assent are submitted, Qualtrics dispatches the baseline survey (T1) to the email addresses provided.

The T1 survey takes 10–20 min for parents/guardians and 45–60 min for young people. A progress bar will be included to help participants track their completion. To encourage participation, automated reminder emails will be sent at 5‐day intervals if surveys are not submitted. Participants will receive three email reminders (on days 5, 10, and 15), each containing a survey link and an opt‐out link. If participants do not complete the survey after these reminders, the research team will call the participant using the number provided at consent.

Participants will be provided options to complete the survey if they face difficulties with the online version. For example, participants will be offered a paper version sent by post, or have their data collected over the telephone. Participants who do not submit their T1 survey within 1 month will be marked as deviating from the study protocol and their data will be handled separately in a sensitivity analysis.

At the 6‐month follow‐up (T2), participants will receive a new survey invitation via email. The T2 survey will take 5–10 min for parents/guardians and 20–30 min for young people. Automated reminders and follow‐up procedures will mirror those at T1. The final survey (T3) will occur 12 months after the initial baseline (T1). The T3 survey will take 5–10 min for parents/guardians and 30–40 min for young people. As with previous timepoints, automated reminders and follow‐up calls will be sent.

Participants will be compensated for completing the surveys using electronic vouchers (https://www.voucherexpress.co.uk/giftcard/vex‐gift‐card.aspx). Parents/guardians can receive up to £60 in e‐vouchers, with £20 awarded for each timepoint completed. Young people can earn up to £75, including £10 for each completed survey and bonuses of up to £45 for submitting surveys within 1 month of receiving the link. The bonuses will include £5 for completing the T1 survey and £20 for each timely submission of the T2 and T3 surveys. Bonuses will be paid at the end of T3. Participants who do not complete surveys on time are ineligible for bonus payments.

At the end of each survey, signposting information will be provided. If a young person endorses a response that raises a safeguarding concern, the research team will be alerted via automated email. A review of the young person's data will be conducted and handled on a case‐by‐case basis in consultation with the senior author (ES).

### Sample size

The sample will consist of 600 young people from year 7 (ages 11/12), year 8 (ages 12/13), year 9 (ages 13/14), year 10 (ages 14/15) and year 11 (ages 15/16). See Table 1 for the breakdown split by year group and by recruitment source.

To evaluate our primary research question—whether ADHD and autism traits, and/or their interaction at T1 predict an increase in self‐reported depression symptoms at T3—a sample size of 528 is required. This sample size allows detection of a small but clinically meaningful effect size of *f* ^2^ = 0.025 with 90% power and an alpha level of 0.05, using multiple regression in *G*Power 3.1.7 (Faul et al., 2009). This corresponds to a partial *R* ^2^ of 2%, representing the additional variance in depression symptoms explained by any one predictor (ADHD, autism, or their interaction), conditional on the inclusion of five additional predictors in the model. Notably, reducing the alpha level to 0.01 would still allow detection of effect sizes of *f* ^2^ = 0.025 (partial *R* ^2^ = 2.5%) with adequate power.

Sample size calculations for complex cross‐lagged models remain challenging, even when simulation‐based approaches are used, as they require specifying numerous unknown parameters. However, for a simple mediation path, a sample size of 600 provides 90% power to detect an indirect effect when both the exposure‐mediator and mediator‐outcome path has partial correlations of *r* = 0.15 (equivalent to a partial *R* ^2^ of 0.0255), using the R package pw2ppl (Aberson, 2019). This reflects the unique contribution of each path while adjusting for covariates in the model.

## ANALYSIS

Participant data submitted outside of the specific timepoint window will be analysed separately in a sensitivity analysis.

### Descriptives

Descriptive statistics, including mean, standard deviation, median, minimum and maximum for numeric variables, as well as frequencies (%) for categorical variables, will be presented for each variable at each timepoint (T1, T2 and T3).

### Main analysis

The analysis plan will be structured to address the research questions. Prior to the main analyses, baseline associations between ADHD, autism and mediators and moderators will be calculated.

First, to assess our first primary research question, whether high levels of ADHD, autism, and their interaction are associated with depressive symptoms at 6 and 12 months, adjusting for baseline depression levels, linear multilevel regression analyses will be performed. ADHD and autism at baseline, along with their interaction (ADHD × Autism), Time (treated as a categorical variable) and baseline depression, will be included as fixed effects. A random intercept will be specified for each participant to account for the repeated measures design. The model will also examine whether the effects of autism and ADHD on depressive symptoms vary across timepoints by incorporating a three‐way interaction (Autism × ADHD × Time). To account for within‐subject correlation, an unstructured covariance matrix will be used, allowing residual variances and covariances to differ across time points. “School” will be included as a random effect to account for clustering within schools, and the necessity of this will be assessed for more complex future analyses. The model will be estimated using Restricted Maximum Likelihood (REML).

In the next step, the potential confounding effect of age will be controlled, and the interaction between age and time will be analysed to capture developmental changes. In a second step, additional covariates will include sex, cognitive ability, SES, and academic term (termed ‘season’) when data will be collected.

To assess whether high baseline levels of ADHD, autism, and their interaction are associated with increased EB, specifically higher CUE frequency and intensity, and ERD at T2 and T3, similar mixed‐effects linear regressions will be performed for each outcome. Sensitivity analyses will be undertaken to examine the effects of controlling for medication status.

Moving on to our second primary research questions — whether elevated EB and/or ERD at T2 mediate the pathway from baseline ADHD, autism, and their interaction to increased depression symptoms at T3 ‐ a longitudinal cross‐lagged mediation panel model (CLPM; Little et al., 2007) will be conducted (see Figure 2). This model, utilising three waves of data (T1, T2, and T3), will include autoregressive paths between the same variables over time to control for their stability, as well as cross‐lagged effects where a variable at time *t* influences a different variable at time *t*+1, accounting for temporal relationships and potential reciprocal effects. The data for the CLPM will be organised in a wide format and analysed as a path model within structural equation modelling. All continuous variables will be mean‐centred for ease of interpretation, especially in cases involving interactions. This approach to estimate the indirect effect of ADHD, autism, and their interaction at T1 on depressive symptoms at T3 via the mediator EB at T2 by multiplying the cross‐lagged path coefficients from ADHD, Autism and their interaction at T1 to EB at T2, and from EB at T2 to Depression symptoms at T3. If the interaction is neglectable (i.e., *p* > 0.1), it will be removed for easier interpretation. Other mediating pathways (i.e., with ERD as the mediator) will be estimated similarly. If an indirect path involving the interaction between ADHD and autism exists, the indirect paths of autism and ADHD will be presented at the mean levels of these two variables, providing clarity on their individual and combined contributions. The secondary analyses with parent‐observed depression and anxiety symptoms will be conducted in a similar way as the CLPM. Given their known overlap with anxiety, and oppositionality and defiance, we will examine the specificity of any ERD/EB mediating effects observed by running sensitivity analyses in which these variables are included as additional mediators.

In the sensitivity analyses, we will control for baseline confounders (age, sex, cognitive ability, SES and academic term [‘season’]) in our cross‐lagged model by regressing all subsequent waves of predictors, mediators and outcome on each confounder. This will help reduce bias in estimating indirect effects. However, including multiple covariates in this way can lead to convergence and estimation issues in cross‐lagged models. In such cases, we may have to adopt a more parsimonious approach by regressing only the primary outcomes Y at waves 2 and 3 on the baseline confounders. This effectively adjusts the *X*_1_→Y_2_ and *X*_1_→Y_3_ paths, which are of primary interest for our research questions.

The standard CLPM is well suited to answer our primary mediational question as it captures between‐person (average) effects over time and is manageable given our study's complexity, with multiple predictors, mediators and moderators across only three timepoints. The key assumption of the CLPM that the effects will be fixed will be relaxed by including random effect. While the Random Effects CLPM (RE‐CLPM) is conceptually appealing because it separates between and within person variances, it often requires more timepoints, simpler model structures of larger samples to converge and produce reliable and interpretable estimates.

To assess the robustness of our findings, we will conduct sensitivity analyses using RE‐CLPMS (Hamaker et al., 2005). We will focus on simplified versions of the model targeting central hypothesised pathways (e.g., ADHD ‐ > EB ‐ > depression). By fitting this core structure in both the standard CLPM and RE‐CLPM frameworks, we can compare the magnitude and direction of path estimates and evaluate whether key associations hold when accounting for potential within‐person dynamics.

If path estimates are consistent across CLPM and RE‐CLPM, this would strengthen confidence in our conclusions. However, if the models diverge—particularly in the direction or statistical significance of key paths—we will interpret these findings with caution and clarify in the manuscript that conclusions about these associations may be model‐dependent.

All analyses will be performed using structural equation modelling (SEM) with Full Information Maximum Likelihood (FIML) for model estimation. FIML handles missing data under the assumption that data will be Missing at Random (MAR). We will assess potential bias due to missing data by comparing baseline variables between participants with and without missingness. If systematic differences are detected, we will include predictors of missingness, if not already part of the model, as auxiliary variables to improve estimation. These auxiliary variables will be specified in a way that does not influence the structural part of the model (Graham, 2003) but further increase the likelihood of MAR. Standard errors and confidence intervals for indirect effects will primarily be estimated using bootstrapping. If missing data is substantial, parametric methods such as the delta method will be employed instead. The model fit of the CLPM will be evaluated using standard indices, including the Comparative Fit Index (CFI), Tucker‐Lewis Index (TLI), and Root Mean Square Error of Approximation (RMSEA). A good fit will be indicated when CFI and TLI are both ≥0.90, and RMSEA is ≤ 0.05, with values up to 0.08 considered fair.

These fit indices will not be available for the RE‐CLPM. Comparisons between models (i.e., models with and without interactions) and between the CLPM and the RE‐CLPM will be based on the Akaike Information Criterion (Mund & Nestler, 2019). This comprehensive approach will allow for determining whether elevated EB and/or ERD at T2 will mediate the relationship between baseline ADHD, autism, and their interaction, and increased depression symptoms at T3, and whether the observed effects will be consistent across within‐ and between‐person levels.

### Exploratory analysis (research questions 3–6)

Exploratory analyses will address Research Questions 3–6 using the same modelling approach (CLPM) to estimate the proposed direct, indirect, and moderating effects linking baseline ADHD and autism traits to depression symptoms over time. Specifically, we will examine whether low self‐esteem at T2 will mediate the relationship between baseline ADHD, autism, EB, and/or ERD to depression symptoms at T3 (Research question 3; Figure 3). Additionally, individual differences in resilience and rumination at T1 will be tested as potential moderators of the pathway from baseline ADHD, autism, and their interaction to depression symptoms at T3 (Research question 4; Figure 4) as single moderators. The temporal relationships between baseline ADHD, autism, and EB at T1, to ERD at T2, and depression symptoms at T3 (Research question 5; Figure 5) will also be analysed. To compare models featuring primary and secondary outcomes (Research Question 5), we will fit a model that includes the secondary outcome and employ both the Akaike Information Criterion (AIC) and Bayesian Information Criterion (BIC) for model comparison, see below. These analyses will aim to clarify the mediating and moderating mechanisms underlying the progression from ADHD and autism‐related traits to depression symptoms, providing insights into the temporal dynamics involved. A detailed statistical analysis plan, including specific guidelines and generic code for conducting the exploratory questions, will be finalised before data collection concludes to ensure transparency and consistency in the analyses.

### Model selection and evaluation

Our research questions will be assessed by the overall model fit, model comparison using information criteria (ICs) and statistical tests of specific pathways. Model comparisons will be guided by ICs, specifically the Akaike Information Criteria (AIC) and the Bayesian Information Criteria (BIC), following Stahl et al. (2014). We will compare increasingly complex, prespecified models to evaluate whether the added complexity improves model fit meaningfully or results in overfitting. To support interpretation, we will report evidence ratios such as BIC weights to quantify relative support for competing models (Burnham & Anderson, 2004).

The final selected model will then be examined using statistical inference. Pre‐specified hypotheses will be tested at an alpha level of 0.05. For exploratory non‐prespecified hypotheses, Hochberg's False Discovery Rate (FDR) correction will be applied within each model to control the proportion of false positives among significant results, at 5%. Because FDR correction depends on the distribution of *p*‐values within a given model, it is not possible to report corrected *p*‐values directly. Instead, we will transparently report which *p*‐values survive the FDR threshold.

If the overall model fit is acceptable, some non‐significant paths may still be retained if they are theoretically justified. All analysis decisions and any post‐hoc model modifications will be documented and made publicly available in a GitHub library to support transparency and open science.

At the time of this submission, T1 data collection is complete and T2 data has begun. T1 data has been included in an analysis of the MESI to validate its psychometric properties (Lukito et al., in press).

## DISCUSSION

The findings from the MEMO study will inform other studies within the RE‐STAR programme, aiding the development of a school‐based preventative intervention. The analyses will help inform the active components of this intervention. For instance, if EB is found to mediate the pathways between neurodivergence and depression symptoms, then we will seek to develop ways to reduce the exposure to CUEs and ameliorate the intensity by which NYP respond to them.

## AUTHOR CONTRIBUTIONS

**Edmund J. S. Sonuga‐Barke**: Conceptualization; funding acquisition; methodology; project administration; writing—original draft; writing—review and editing. **Melanie Palmer**: Conceptualization; writing—original draft. **Kirsty Griffiths**: Conceptualization; writing—review and editing. **Anna Wyatt**: Writing—original draft. **Andrea Danese**: Conceptualization; funding acquisition; methodology; writing—original draft; writing—review and editing. **Susie Chandler**: Conceptualization; methodology; project administration; writing—original draft; writing—review and editing. **Daniel Stahl**: Conceptualization; formal analysis; funding acquisition; writing—original draft; writing—review and editing. **Steve Lukito**: Conceptualization; writing—review and editing. **Georgia Pavlopoulou**: Conceptualization; writing—review and editing. **Emily Simonoff**: Conceptualization; funding acquisition; methodology; writing—original draft; writing—review and editing.

## CONFLICT OF INTEREST STATEMENT

The authors declare no conflicts of interest.

## ETHICAL CONSIDERATIONS

The study was approved by NHS Health and Social Care Research Ethics Committee (HSC REC A; REC reference 23/NI/0090) and will be conducted in accordance with the Declaration of Helsinki. Informed parental consent and young person assent will be obtained from for all participants prior to their participation.

## CODE AVAILABILITY STATEMENT

At the end of the study, code from the study, including scoring and analysis scripts, will also be made available through the UK Data Service.

## Supporting information

Supporting Information S1

## Data Availability

At the end of the study, anonymised datasets from this study will be made available through the UK Data Service. Anonymised datasets will also be available, upon reasonable request from the senior author (E. Simonoff).
