# Supplementary material for: Why do attention‐deficit/hyperactive disorder and/or autism traits place adolescents at risk for depression? Protocol for a longitudinal comparison of the mediating role of emotion regulation deficits versus emotional burden
Source: JCPP Adv. 2025 Oct 28;6(1):e70052. doi: 10.1002/jcv2.70052 (PMC12973166; doi:10.1002/jcv2.70052)
Supplement: Supplementary file 1 — Supporting Information S1 [file JCV2-6-e70052-s001.docx]

**Supporting Information I:**

**Table S1: Design Summary for Primary Research Questions**

|  | Question | Hypothesis | Sampling plan | Analysis Plan | Rationale for deciding the sensitivity of the test for confirming or disconfirming the hypothesis | Interpretation given different outcomes | Theory that could be shown wrong by the outcomes |
| --- | --- | --- | --- | --- | --- | --- | --- |
| **Primary Research Questions & Analysis** | | | | | | | |
| 1 | Are ADHD and/or autism traits (T1) associated with later depression symptoms and ERD and/or EB (at T2 and T3 controlling for baseline outcome levels)? | Adolescents with higher ADHD/autism traits at T1 will show greater levels in depression symptoms and higher ERD and EB at T2 and T3, controlling for baseline levels. | 600 adolescents (ages 11–16), enriched for ADHD/autism diagnoses, recruited via schools, NHS clinics and charities. Stratified by age and sex | Linear multilevel regression analyses with ADHD and autism traits (and their interaction), time, and baseline depression as predictors. Random intercepts for participants; "school" as random effect. Covariates: age, sex, IQ, SES, academic term. | N=600 powered for effect sizes with 90% power at α=0.05. | Significant results suggest ADHD/autism traits drive increases in depression symptoms, ERD and EB change. Null findings imply trait levels alone may not explain the emergence of depression symptoms. | ADHD and/or autism traits place adolescents at greater risk for later depression symptoms, ERD and EB. |
| 2 | Q2. Do ERD and/or EB (T2) mediate the developmental association between ADHD and/or autism (T1) and depression symptoms (T3)? | ERD and/or EB at T2 mediate the association between ADHD/autism traits at T1 and depression symptoms at T3 | Same as above. | Longitudinal cross-lagged panel model (CLPM) estimating autoregressive and cross-lagged paths between ADHD/autism, ERD/EB, and depression. SEM with FIML for missing data; bootstrap CIs for indirect effects | Sample size (N=600) provides 90% power to detect indirect effects. Sensitivity to within- vs between-person effects assessed using RE-CLPM | Significant mediation supports ERD/EB as potential intervention mechanisms. Non-significant mediation suggests alternative pathways may drive the pathway from ADHD/autism traits to depression. | EB hypothesis: emotional burden from CUEs (frequency × intensity) mediates ADHD/autism-depression link.  ERD hypothesis: emotion regulation deficits mediate ADHD/autism-depression link. |

**Supporting Information II: Additional measures collected in MEMO**

**Additional measures to be completed by the young person.**

**Depression symptoms:** The Patient Health Questionnaire (PHQ-A, Johnson, J. G. et al., 2002) is a 9-item self-report measure that asks about a young person’s mood in terms of frequency in the past 2 weeks. The PHQ-A uses a 4-point Likert scale ranging from 0 (*I*) to 3 (*Nearly every day*). Higher scores indicate greater depressive symptoms. The PHQ-A is a standardised measure to be collected by research programmes funded by the Adolescence, Mental Health and Developing Mind initiative, as recommended by the funders.

**Anxiety symptoms:** The Generalised Anxiety Disorder (GAD-7, Spitzer et al., 2006) is a 7-item self-report questionnaire asking how young people have been feeling in the last 2 weeks. The GAD-7 uses a 4-point Likert scale ranging from 0 (*Not at all*) to 3 (*Nearly every day*). Higher scores indicate greater anxiety symptoms. The GAD-7 is widely used in research and clinical settings, has good accuracy with adolescent samples (Mossman et al., 2017). The GAD-7 s a standardised measure to be collected by research programmes funded by the Adolescence, Mental Health and Developing Mind initiative, as recommended by the funders.

**Post-Traumatic Stress Disorder symptoms:** The Child PTSD Symptoms Scale self-report (CPSS-SR, Foa et al., 2018) is a 20-item self-report questionnaire that will assess post-traumatic symptoms. The CPSS-SR will ask about 20 specific events that may have occurred in the past, and for any that endorsed will be followed up with an item on its severity. Severity will be rated on a 4-point scale, ranging from 0 (*Not at all*) to 4 (*Almost always*). Scores will range from 0-80, with higher scores indicating greater PTSD symptom severity.

**Digital stressors and hassles:** The Digital Activity, Feelings and Impact (DAFI) is a 15-item self-report questionnaire to be completed by young people. The DAFI was developed by Sonuga-Barke and colleagues (Kostyrka-Allchorne et al., 2025) as part of another research project to assess a young person’s online activity in the past 2 weeks. Initial validation shows two factors that are associated with risk: content risk (7-items) and conduct risk (8-items). Items will be rated on a 4-point Likert scale, ranging from 0 (*Never*) to 3 (*Everyday*).

**Loneliness:** The Roberts UCLA Loneliness Scale (Roberts et al., 1993), is a 4-item self-report measure to be completed by young people. Four items assessing the subjective feelings of loneliness and social isolation will be rated on a 4-point Likert scale ranging from 1 (*Never*) to 4 (*Often*). The UCLA has been validated for use with adolescent samples. Higher scores will represent higher levels of loneliness.

**Positive wellbeing:** The Short-version Warwick Edinburgh Mental Wellbeing Scale (SWEMWBS, NHS Health Scotland, University of Warwick and University of Edinburgh, 2008) is a 7-item self-report measure to be completed by young people. The SWEMWBS asks about positive wellbeing in the previous 2 weeks, and is rated on a 5-point Likert scale ranging from 1 (*Not of the time*) to 5 (*All of the time*). The SWEMWBS has been validated with young people aged 15-21 years (McKay & Andretta, 2017). Higher scores indicate greater positive wellbeing.

**Generic daily stressors and hassles:** The Adolescent Perceived Events Scale (APES, Compas et al., 1987) is a 90-item self-report questionnaire completed by young people to assess the frequency and perceived impact of life events. Developed to capture a comprehensive range of both positive and negative occurrences, the participant indicates whether they have experienced the event in the past 6-months. If so, the young person will then rate their perceived desirability of that event, which will be rated on a 9-point Likert scale ranging from -4 (*Extremely bad*) to 4 (*Extremely good*). The APES can be scored in a variety of ways, including calculating the total weighted scores for both negative and positive events, which can be categorised according to whether the event is a daily hassle or a major life event.

**Stress Perception**: The Perceived Stress Scale-10 (Cohen & Williamson, 1988) is a 10-item self-report measures and is a shortened version of the original PSS (Cohen et al., 1983). The PSS-10 evaluates the degree to which individuals perceive their life as stressful. Young people rate feelings and thoughts in the past month, using a 5-point scale ranging from *never* (1) to *very often* (5). Total PSS-10 scores range from 10 to 50, with higher scores indicating greater perceived stress. PSS-10 has been validated in adolescent populations (Steen et al., 2020; Whitney et al., 2022) and in autistic population (Bishop-Fitzpatrick et al., 2018; Thoen et al., 2023) as well as those with ADHD symptoms (Whitney et al., 2022)

**Additional measures rated by parent/guardian**

**Emotional regulation:** A parent-guardian version of the DERS (DERS-P; Bunford et al., 2020) is a 18 item measure based on the observed behaviours from the child. The DERS-P asks the parent/guardian to rate their emotional regulation, using a five-point Likert scale ranging from 1 (*Almost never*) to 5 (*Almost always*).

**Engagement and confidence in school:** The Wider Outcomes Survey for Parents (WOSP; Humphrey & Squires, 2011) is an 8-item measure that will assess how well the school engages with parents/guardians of young people with SEN and parental confidence in the school. Each item will be rated on a 4-point Likert scale, ranging from 1 (*Strongly disagree*) to *4 (Strongly agree).* Higher total score represents greater parent-perceived engagement and confidence in a school.

**Supporting Information III: The My Emotions in School Inventory (MESI)**

The MESI contains 25 commonly upsetting events (CUEs) (Table S1). Each CUE is rated on five-point Likert scales on (a) the frequency of upsetting events (i.e., ‘frequency’ variable [0=never, 1=rarely, 2=sometimes, 3=often, 4=frequently]), and (b) how much upset each event would cause (i.e., ‘intensity’ variable [0=not at all, 1=a little, 2=somewhat, 3=a lot, to 4=extremely]). The frequency and intensity variables are shown for the first CUE only in Table S1, as an example.

**Table S2. My Emotions at School Inventory**

| Here is a list of things that could happen at school that might be upsetting for children. Can you tell us (a) how often this has happened to you, and (b) how upset you would be if it happened today? | | | | | |
| --- | --- | --- | --- | --- | --- |
| 1. | Finding out your peers have been talking about you behind your back. | | | | |
|  | Has this happened to you? | | | | |
|  | Never | Rarely | Sometimes | Often | Frequently |
|  |  |  |  |  |  |
|  | If this happened today, how much would it upset you? | | | | |
|  | Not at all | A little | Somewhat | A lot | Extremely |
|  |  |  |  |  |  |
| 2. | Unexpectedly having to wait for ages in a queue. | | | | |
| 3. | Being told off by your teacher in front of your classmates. | | | | |
| 4. | Schoolmates don’t listen to what you say or ignore you. | | | | |
| 5. | School staff don’t listen to you or challenge what you say. | | | | |
| 6. | School staff don’t understand your feelings and reactions. | | | | |
| 7. | The person in charge makes a last-minute change of plan. | | | | |
| 8. | Not being able to do a task at school. | | | | |
| 9. | Being in a chaotic classroom and/or playground (e.g., too noisy, too many people around you, too many visuals). | | | | |
| 10. | When you’re asked to do something really boring. | | | | |
| 11. | Being made to stop doing something you really enjoy by school staff. | | | | |
| 12. | The sights, smells, or sounds in the classroom make you feel uncomfortable. | | | | |
| 13. | Losing or forgetting something important for your lessons (e.g., your school bag or physical education [PE] kit). | | | | |
| 14. | Getting into trouble for losing and/or forgetting your stuff (e.g., homework). | | | | |
| 15. | Being rushed to complete some work. | | | | |
| 16. | Not ‘getting’ or understanding what others are talking about. | | | | |
| 17. | School staff treating you unfairly (e.g., by giving an unnecessary detention). | | | | |
| 18. | Being teased and/or bullied by peers. | | | | |
| 19. | Others telling you to try harder when you’ve already tried your best. | | | | |
| 20. | Being accused of something you didn’t do. | | | | |
| 21. | Not being able to get something ‘quite right’ (e.g., a drawing, piece of schoolwork, computer game). | | | | |
| 22. | Not being allowed to do the things that help you manage your emotions (e.g., use fidget toys, get out of your seat, leave the classroom). | | | | |
| 23. | Feeling pressure to do well (e.g., in exams, getting homework done, getting a high school grade). | | | | |
| 24. | Having too many options to choose from. | | | | |
| 25. | Being rushed to move from one thing to another. | | | | |

**References**

Bishop-Fitzpatrick, L., Mazefsky, C. A., & Eack, S. M. (2018). The combined impact of social support and perceived stress on quality of life in adults with autism spectrum disorder and without intellectual disability. *Autism*, *22*(6), 703-711.

Bunford, N., Dawson, A. E., Evans, S. W., Ray, A. R., Langberg, J. M., Owens, J. S., ... & Allan, D. M. (2020). The difficulties in emotion regulation scale–parent report: A psychometric investigation examining adolescents with and without ADHD. *Assessment*, *27*(5), 921-940.

Cohen, S., Williamson, G., Spacapan, S., & Oskamp, S. (1988). The social psychology of health.

Cohen, S., Kamarck, T., & Mermelstein, R. (1983). A Global Measure of Perceived Stress. *Journal of Health and Social Behavior, 24*(4), 385-396. 10.2307/2136404

Compas, B. E., Davis, G. E., Forsythe, C. J., & Wagner, B. M. (1987). Assessment of major and daily stressful events during adolescence: the Adolescent Perceived Events Scale. *Journal of Consulting and Clinical Psychology, 55*(4), 534-541. 10.1037/0022-006X.55.4.534

Foa, E. B., Asnaani, A., Zang, Y., Capaldi, S., & Yeh, R. (2018). Psychometrics of the Child PTSD Symptom Scale for DSM-5 for Trauma-Exposed Children and Adolescents. *Journal of Clinical Child & Adolescent Psychology, 47*(1), 38-46. 10.1080/15374416.2017.1350962

Humphrey, N., & Squires, G. (2011). Achievement for all: National evaluation. Final report.

Johnson, J. G., Harris, E. S., Spitzer, R. L., & Williams, J. B. W. (2002). The patient health questionnaire for adolescents: Validation of an instrument for the assessment of mental disorders among adolescent primary care patients. *Journal of Adolescent Health, 30*(3), 196-204. 10.1016/S1054-139X(01)00333-0

Katarzyna, K. A., Jake, B., Aja, M., Mariya, S., Iqra, A., Amy, B., Eliz, A., Chris, H., Ellen, T., Sonia, L., Sonuga-Barke, E. J. S., & Digital Youth Research Programme (2025). Understanding Youth Online Experiences and Mental Health: Development and Validation of the Digital Activity and Feelings Inventory (DAFI). *International journal of methods in psychiatric research*, *34*(2), e70028. <https://doi.org/10.1002/mpr.70028>

McKay, M. T., & Andretta, J. R. (2017). Evidence for the Psychometric Validity, Internal Consistency and Measurement Invariance of Warwick Edinburgh Mental Well-being Scale Scores in Scottish and Irish Adolescents. *Psychiatry Research, 255*, 382-386. 10.1016/j.psychres.2017.06.071

Mossman, S. A., Luft, M. J., Schroeder, H. K., Varney, S. T., Fleck, D. E., Barzman, D. H., Gilman, R., DelBello, M. P., & Strawn, J. R. (2017). The Generalized Anxiety Disorder 7-item scale in adolescents with generalized anxiety disorder: Signal detection and validation. *Annals of Clinical Psychiatry : Official Journal of the American Academy of Clinical Psychiatrists, 29*(4), 227-234A.

NHS Health Scotland, University of Warwick and University of Edinburgh. (2008). *Short Warwick Edinburgh Mental Well-Being Scale (SWEMWBS)*

Roberts, R. E., Lewinsohn, P. M., & Seeley, J. R. (1993). A Brief Measure of Loneliness Suitable for Use with Adolescents. *Psychol Rep, 72*(3), 1379-1391. 10.2466/pr0.1993.72.3c.1379

Spitzer, R. L., Kroenke, K., Williams, J. B. W., & Löwe, B. (2006). A brief measure for assessing generalized anxiety disorder: the GAD-7. *Archives of Internal Medicine, 166*(10), 1092-1097. 10.1001/archinte.166.10.1092

Steen, P. B., Poulsen, P. H., Andersen, J. H., & Biering, K. (2020). Subjective social status is an important determinant of perceived stress among adolescents: a cross-sectional study. *BMC Public Health*, *20*, 1-9.

Thoen, A., Steyaert, J., Alaerts, K., Evers, K., & Van Damme, T. (2023). A systematic review of self-reported stress questionnaires in people on the autism spectrum. *Review journal of autism and developmental disorders*, 1-24.
